# Engineered Artificial MicroRNA Precursors Facilitate Cloning and Gene Silencing in Arabidopsis and Rice

**DOI:** 10.3390/ijms20225620

**Published:** 2019-11-10

**Authors:** Dandan Zhang, Nannan Zhang, Wenzhong Shen, Jian-Feng Li

**Affiliations:** 1 State Key Laboratory of Biocontrol, Guangdong Provincial Key Laboratory of Plant Resources, MOE Key Laboratory of Gene Function and Regulation, School of Life Sciences, Sun Yat-sen University, Guangzhou 510275, China; 2 Department of Biochemistry, Biophysics and Molecular Biology, Iowa State University, Ames, IA 50011, USA; 3 Guangdong Provincial Key Laboratory of Sugarcane Improvement and Biorefinery, Guangdong Bioengineering Institute, Guangzhou 510316, China

**Keywords:** plant genome, artificial microRNA, gene silencing, Arabidopsis, rice

## Abstract

Plant genome sequences are presently deciphered at a staggering speed, due to the rapid advancement of high-throughput sequencing technologies. However, functional genomics significantly lag behind due to technical obstacles related to functional redundancy and mutant lethality. Artificial microRNA (amiRNA) technology is a specific, reversible, and multiplex gene silencing tool that has been frequently used in generating constitutive or conditional mutants for gene functional interrogation. The routine approach to construct amiRNA precursors involves multiple polymerase chain reactions (PCRs) that can increase both time and labor expenses, as well as the chance to introduce sequence errors. Here, we report a simplified method to clone and express amiRNAs in Arabidopsis and rice based on the engineered Arabidopsis miR319a or rice miR528 precursor, which harbor restriction sites to facilitate one-step cloning of a single PCR product. Stem-loop reverse-transcriptase quantitative PCR (RT-qPCR) and functional assays validated that amiRNAs can be accurately processed from these modified precursors and work efficiently in plant protoplasts. In addition, Arabidopsis transgenic plants overexpressing the modified miR319a precursor or its derived amiRNA could exhibit strong gene silencing phenotypes, as expected. The simplified amiRNA cloning strategy will be broadly useful for functional genomic studies in Arabidopsis and rice, and maybe other dicotyledon and monocotyledon species as well.

## 1. Introduction

With the advent of whole-genome sequencing technologies, plant genomic data are expanding at an explosive rate. In the post-genomic era, analyzing these genomic data and studying the functions of newly discovered genes is critical for understanding the nature of plant genomes and accelerating the process of crop improvement. One of the most frequently used strategies to study gene function is to create loss-of-function mutants. In past decades, a large number of mutant libraries in model plant species, such as Arabidopsis and rice, have been constructed through physical, chemical, or biological (T-DNA and transposon insertion) mutagenesis [1,2,3]. However, tedious large-scale screening is required to identify the genes responsible for desired mutant phenotypes [4]. Additionally, random mutagenesis could not broadly cover the whole plant genome. Recently, the powerful CRISPR/Cas9 technology, which enables targeted genome modifications, has already revolutionized plant genome research [5]. Although the CRISPR/Cas9 system is simple, efficient, and highly specific, there are still some limitations related to its application in plant research. First, CRISPR/Cas9-mediated gene disruption is less efficient in targeting heterochromatic regions [6], limiting the range of targetable genes. Second, permanent deletion of essential genes by CRISPR/Cas9 can result in lethality [7,8]. Third, transcripts of many plant genes undergo alternative splicing (AS) in the same or different cell types, producing multiple proteins with different structural domains [9]. However, the CRISPR/Cas9 system is unable to specifically inactivate a certain AS isoform in a cell type-specific manner.

MicroRNAs (miRNAs), a class of endogenous small noncoding RNAs with the size of 21–24 nucleotides, can mediate post-transcriptional and translational gene regulation. miRNAs play important roles in diverse aspects of plant development and plant responses to biotic and abiotic stresses [10,11]. The biogenesis of miRNA is a multistep process that begins with the transcription of a miRNA gene into a primary transcript (pri-miRNA) [12]. Pri-miRNA is sequentially processed into a stem-loop structured precursor (pre-miRNA) by DICER-LIKE1 (DCL1), and pre-miRNA is then processed into miRNA/miRNA* duplex and stabilized by methyltransferase HUA ENHANCER1 (HEN1) [13]. The methylated miRNA duplex is eventually loaded into the ARGONAUTE (AGO) protein to form the so-called RNA-induced silencing complexes (RISCs), followed by the release and degradation of miRNA* [14]. By targeting complementary sequences, RISCs negatively regulate gene expression through mRNA degradation and/or translation inhibition [14,15].

Artificial microRNA (amiRNA) technology has already been successfully developed to silence target gene expression by producing artificially designed miRNAs using the naturally existing miRNA precursor as a backbone [16,17]. Compared to genome editing tools, the amiRNA technology offers more flexibility and reversibility in generating loss-of-function mutants without altering DNA sequences. Since the expression of amiRNAs can be tightly controlled by chemical-inducible or cell/tissue-specific promoters [17], amiRNAs are widely utilized for investigating gene functions associated with mutant lethality [18,19]. Moreover, amiRNA has a high silencing specificity and only recognizes target sequences with less than 5 mismatches [17,20], making it an ideal tool to silence individual AS isoforms or multiple genes sharing short conserved sequences [17,21].

In general, amiRNA-expressing plasmids are constructed according to the method described by Schwab et al. [17], as follows: The miRNA and miRNA* of pre-miR319a are replaced by amiRNA/amiRNA* sequences through site-directed mutagenesis using overlapping polymerase chain reactions (PCRs). However, this method is time-consuming and cost ineffective because it involves four PCRs using three pairs of primers. Here, we report a simplified method for amiRNA cloning. We modified the most commonly used miRNA precursor backbones, pre-miR319a for Arabidopsis or related dicot species, and pre-miR528 for rice or related monocot species, by introducing restriction sites using PCR. With the modified amiRNA backbones, only one PCR is needed to amplify the stem-loop fragment containing a newly designed amiRNA/amiRNA* duplex with restriction enzyme sites, which can then be easily inserted into the engineered pre-miR319a or pre-miR528 in the expression vectors. We also provided evidence that amiRNAs produced in this way can be equally effective in protoplasts or transgenic plants as those produced using the traditional approach.

## 2. Results

### 2.1. Strategy for Simplified amiRNA Construction Using a Modified Arabidopsis miRNA319a Backbone

**Figure 1:**
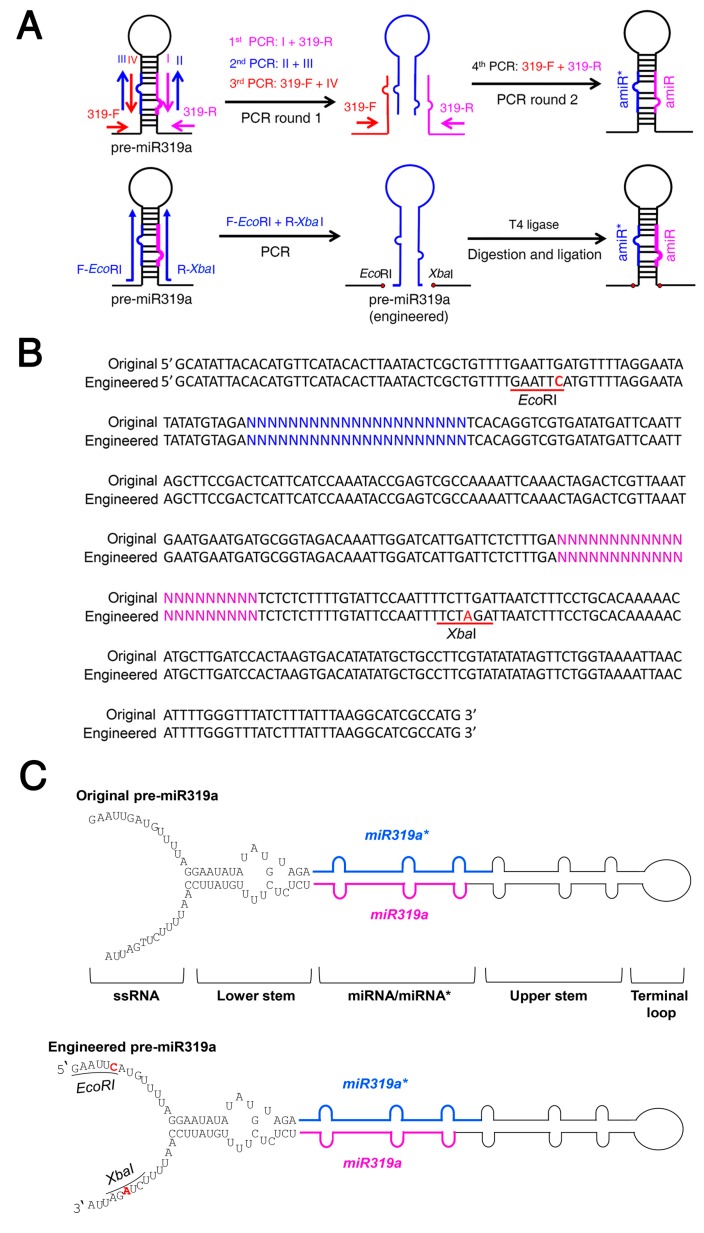
Engineered Arabidopsis miR319a precursor allows a one-step construction of a new amiRNA precursor. (**A**) Diagram of amiRNA construction using the engineered miR319a precursor (pre-miR319a). The upper diagram describes an overlapping PCR strategy for generating a new amiRNA precursor by 4 PCRs in two rounds. In the first round, 3 independent PCRs are performed using the indicated primers. A mixture of 3 PCR products is used as a template to conduct the second round of PCR (4^th^ PCR) using the indicated primers. The lower diagram describes a restriction enzyme-based strategy to assemble a new amiRNA precursor. The *Eco*RI/*Xba*I sites are created at the base of miR319a stem-loop in the engineered pre-miR319a. A single PCR product amplified using a pair of mega-primers containing customized amiRNA/amiRNA* sequences, and *Eco*RI/*Xba*I sites are digested by *Eco*RI/*Xba*I and inserted into the same digested engineered pre-miR319a. In the resulting amiRNA precursor, the amiRNA and amiRNA^*^ are colored in magenta and blue, respectively. (**B**) The engineered pre-miR319a contains a G-to-C mutation and a T-to-A mutation that create *Eco*RI and *Xba*I sites (underlined), respectively. The nucleotides in magenta and blue correspond to amiRNA and amiRNA^*^, respectively. (**C**) Diagram of the original and engineered pre-miR319a. Mutated nucleotides are highlighted in red.

The previous overlapping PCR strategy to assemble a new amiRNA precursor involves four PCRs in two rounds (Figure 1A) [17]. To simplify the procedure and accelerate the amiRNA construction process, we tried to engineer the pre-miR319a backbone by introducing minor changes in its DNA sequences to create restriction sites for amiRNA sequence insertion (Figure 1A–C). For many plant miRNA precursors, the lower stem located ~15 nt below miRNA/miRNA* is critical for miRNA processing. A single change in the lower stem of pre-miRNA can completely abolish miRNA processing [22,23,24]. The ssRNA (single strand RNA) region, an unpaired region downstream of the lower stem seems to be less important for miRNA production [23,25]. Thus, we modified the pre-miR319a backbone by mutating GAATTG and TCTTGA sequences within the ssRNA region to *Eco*RI (GAATTC) and *Xba*I (TCTAGA) restriction sites, respectively (Figure 1B,C). After the modifications, a single PCR product of the stem-loop fragment containing the amiRNA/amiRNA* sequences can be inserted into the amiRNA backbone (Table S1) using *Eco*RI/*Xba*I, which greatly simplifies the construction procedure and enables possible high-throughput amiRNA construction.

### 2.2. Engineered Pre-miR319a Generated Functional miR319a as Demonstrated by the Silencing Phenotype

**Figure 2:**
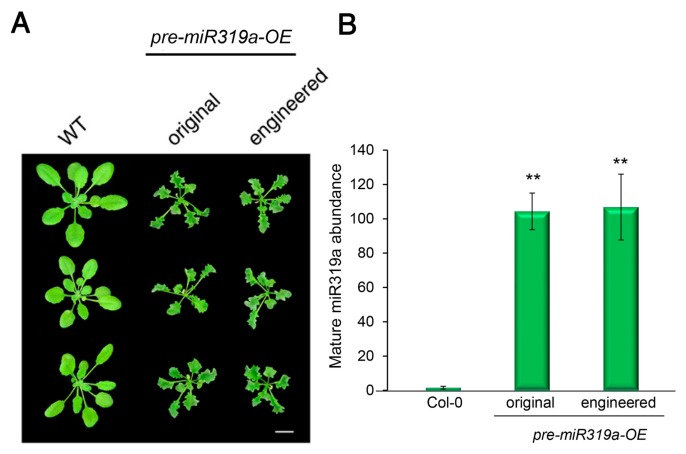
Engineered pre-miR319a retains its function in transgenic plants. (**A**) Phenotypic comparison of transgenic Arabidopsis plants expressing the original or engineered miR319a precursor. Four-week-old plants are shown. Scale bar, 0.5 cm. (**B**) Stem-loop RT-qPCR validates comparable production of mature miR319a from the original or engineered pre-miR319a in transgenic plants. The quantitative PCR data are presented as means ± SD of at least three independent repeats with endogenous *snoR101* expression level set as 1. ** *p* < 0.01 (student’s *t* test).

To test whether the engineered pre-miR319a remains functional, we generated Arabidopsis transgenic plants overexpressing the original and engineered pre-miR319a, respectively. It has been previously reported that miR319a controls Arabidopsis leaf development and morphogenesis through targeting and down-regulating the expression of several TCP (Teosinte branched1/Cycloidea/Proliferating cell factor) family members [26,27,28,29]. Arabidopsis gain-of-function mutant *jaw-D* with overexpression of miR319a exhibits a jagged and wavy leaf phenotype [26]. As expected, the transgenic plants overexpressing both the original and engineered pre-miR319a showed a curly and serrated leaf phenotype (Figure 2A). The relative abundances of mature miR319a in these transgenic plants were further determined using the stem-loop RT-qPCR technique that is specialized for accurate quantification of mature miRNA [30]. We detected comparable production of mature miR319a from engineered pre-miR319a as original pre-miR319a (Figure 2B). These results suggest that mature miR319a can be generated from the modified pre-miRNA319, as well as from the native pre-miR319a.

### 2.3. amiRNAs Produced from Engineered Pre-miR319a Have Comparable Efficiencies in Gene Silencing

**Figure 3:**
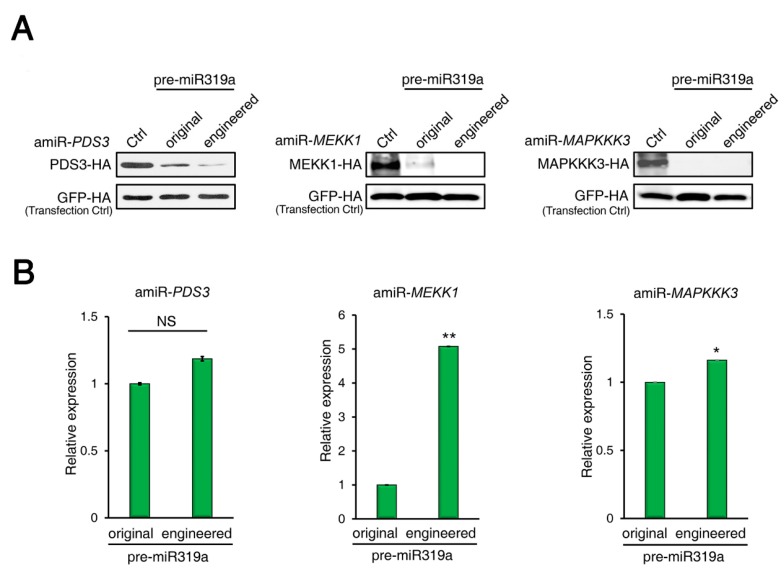
amiRNAs produced from engineered pre-mir319a exhibit equal efficacy in silencing target gene expression. (**A**) Comparison of the performance of amiRNAs produced from the original or engineered pre-miR319a using the ETPamir assay. amiRNAs expressed from engineered pre-miR319a are slightly more effective in silencing Arabidopsis *PDS3*, *MEKK1*, and *MAPKKK3* expression than those expressed from original pre-miR319a in protoplasts. Three independent repeats with GFP-HA as an untargeted internal control produced similar results. (**B**) Detection of mature amiRNAs produced from the original or engineered pre-miR319a in the ETPamir assay. Mature amiRNAs were detected using stem-loop RT-qPCR. The quantitative PCR data represent means ± SD of at least three independent repeats using *mCherry-HA* as a transfection control. * *p* < 0.05, ** *p* < 0.01 (student’s *t* test).

To provide more evidence that the modifications of pre-miR319a would not affect amiRNA processing and maturation, we compared side-by-side the silencing efficiencies of amiRNAs produced from the original or engineered pre-miR319a using an ETPamir assay [31]. In ETPamir assay, a target gene encoding epitope-tagged target protein is co-expressed with individual amiRNAs in protoplasts, and the silencing efficiency of each amiRNA is inversely reflected by the accumulation of target proteins, which can be monitored by immunoblotting using anti-tag antibodies [31,32]. By targeting Arabidopsis *PHYTOENE DESATURASE 3* (*PDS3*), *MAP/ERK KINASE KINASE 1 (MEKK1*) or *MAP KINASE KINASE KINASE 3* (*MAPKKK3*), we found that the amiRNAs produced from the engineered pre-miR319a appeared to be as efficient as or even slightly more effective than those from the original pre-miRNA319a (Figure 3A). We also measured the abundances of mature amiRNAs produced in ETPamir assays by stem-loop RT-qPCR and found that the engineered pre-miR319a could produce comparable or even higher amounts of mature amiRNAs than the original pre-miR319a (Figure 3B). These results imply that the engineered pre-miR319a is fully functional in generating mature amiRNAs.

### 2.4. amiRNAs Produced from Engineered Pre-miR319a Could Effectively Silence Target Gene Expression in Transgenic Plants

**Figure 4:**
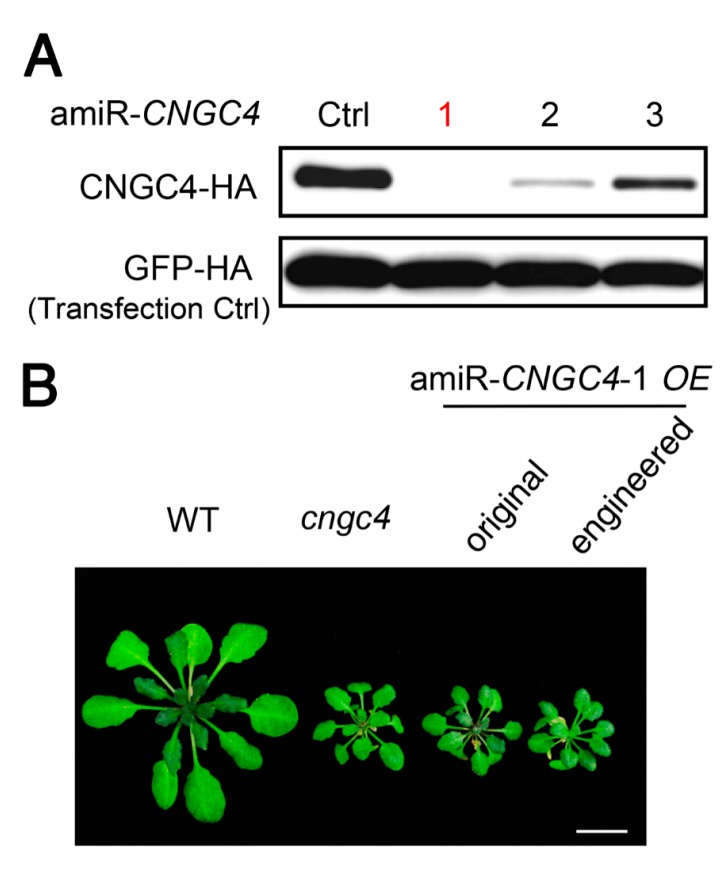
amiRNAs produced from the engineered pre-miR319a are fully functional. (**A**) Comparison of the silencing efficiency of three *CNGC4*-targeting amiRNAs expressed from the engineered pre-miR319a using the ETPamir assay. Note that amiR-*CNGC4*-1 (red) is the most potent amiRNA for silencing *CNGC4*. Three independent repeats with GFP-HA as an untargeted internal control produced similar results. (**B**) Comparison of the performance of amiR-*CNGC4*-1 produced from the original or engineered amiRNA precursor in transgenic plants. *cngc4* is a T-DNA insertion null mutant of *CNGC4*. Scale bar, 0.5 cm.

Next, we evaluated the efficiencies of amiRNAs produced from engineered pre-miR319a in planta. *CNGC4* (CYCLIC NUCLEOTIDE-GATED CATION CHANNEL 4) was selected as the target gene as the null phenotype of *CNGC4* has been reported [33,34] and is easy to observe. Three amiRNAs targeting *CNGC4* were constructed using the engineered pre-miR319a as backbone and their activities were assessed first by the ETPamir assay. The results showed that three amiR-*CNGC4s* could all suppress *CNGC4* expression, but they displayed different silencing efficiencies. amiR*-CNGC4-*1 could almost completely silence *CNGC4* expression (Figure 4A), whereas amiR*-CNGC4-*2 and amiR*-CNGC4-*3 were less effective. So, we chose amiR*-CNGC4-*1 to silence endogenous *CNGC4* in our transgenic plants. The engineered or original pre-amiR-*CNGC4-*1 construct was subsequently introduced into Arabidopsis Col-0 plants. Transgenic plants overexpressing engineered or original pre-amiR-*CNGC4-*1 both exhibited smaller leaves and shorter petioles relative to the wild-type plants, resembling the dwarf phenotype of *cngc4* T-DNA null mutant (Figure 4B). These results validate that the engineered pre-miR319a can be utilized to produce effective amiRNAs for target gene silencing.

### 2.5. Strategy for Simplified amiRNA Construction Using a Modified Rice miRNA528 Backbone

**Figure 5:**
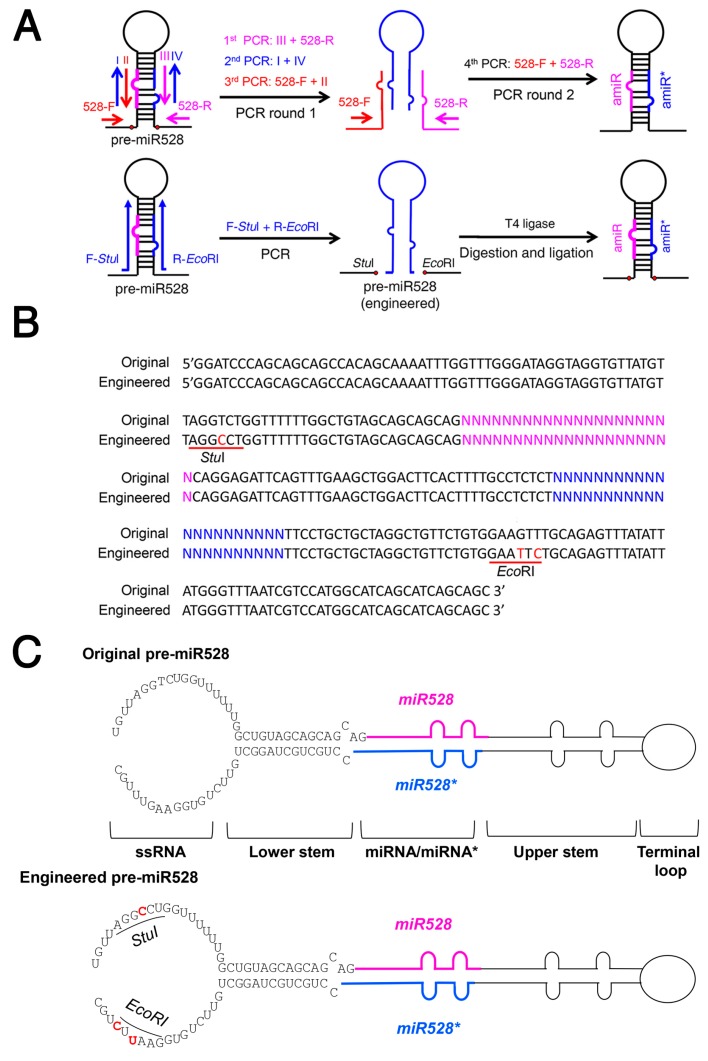
The engineered rice miR528 precursor allows a one-step construction of a new amiRNA precursor. (**A**) Diagram of amiRNA construction using the engineered miR528 precursor (pre-miR528). In the engineered pre-miR528, *Stu*I /*Eco*RI sites are created at the base of miR528 stem-loop. The amiRNA and amiRNA^*^ are colored in magenta and blue, respectively. (**B**) The engineered pre-miR528 contains mutations that can create *Stu*I and *Eco*RI sites (underlined), respectively. The nucleotides in magenta and blue correspond to amiRNA and amiRNA^*^, respectively. (**C**) Diagram of the original and engineered pre-miR528. Mutated nucleotides are highlighted in red.

Rice miR528 precursor (pre-miR528) is frequently used for generating amiRNAs and gene silencing in many monocot species [35,36,37]. To test whether the same strategy can be applied for amiRNA production using pre-miR528, we engineered pre-miR528 by mutating AGGTCT and GAAGTT sequences in the ssRNA region to *StuI* (AGGCCT) and *Eco*RI (GAATTC) restriction sites, respectively (Figure 5A–C). Therefore, PCR products of the stem-loop fragment containing the amiRNA/amiRNA* duplex can be generated using a pair of mega primers (Table S2) and be readily inserted into the engineered pre-miR528 after *Stu*I/*Eco*RI digestion and ligation.

**Figure 6:**
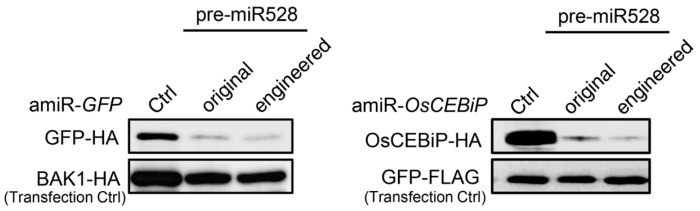
amiRNAs produced from the engineered pre-miR528 are functional in rice cells. Comparison of the performance of amiRNAs produced from the original or engineered pre-miR528 was conducted by the ETPamir assay. Three independent repeats with GFP-FLAG or BAK1-HA as an untargeted internal control produced similar results.

We next evaluated the silencing efficiencies of amiRNAs produced from the engineered pre-miR528 using the ETPamir assay. The amiRNAs produced from both original and engineered pre-miR528 could trigger efficient silencing of the foreign gene *GFP* and the rice endogenous gene *OsCEBiP* (*CHITIN ELICITOR BINDING PROTEIN*) in rice cells (Figure 6). There is no detectable difference in silencing efficiencies between amiRNAs produced from the original or engineered pre-miR528 (Figure 6). These data indicate that the same strategy could be applied to pre-miR528 engineering, which leads to production of functional amiRNAs in rice, but with a simple cloning procedure.

## 3. Discussion

The amiRNA technology is not only a powerful genetic tool for generating loss-of-function mutants in basic plant research, but is also an effective strategy to engineer crops for beneficial agronomic traits [38,39] and enhanced disease resistance against pathogens [40,41,42,43] or pests [44,45]. As amiRNA is produced from an endogenous plant miRNA precursor, and the promoter and terminator of an amiRNA expression cassette can be derived from plants, this technology may raise minimal concerns about introducing foreign genetic elements into engineered crops.

Selecting a suitable miRNA precursor backbone to express amiRNAs is vital for successfully silencing target gene expression. Many plant miRNA precursors such as Arabidopsis miR319a, miR172a, miR395, miR390 and rice miR390, and miR528 have been used as backbones for expressing amiRNAs to confer specific gene silencing [17,35,46,47,48]. The Arabidopsis miR319a and rice miR528 precursors are the most commonly used amiRNA expression backbones that have been widely used to generate loss-of-function mutants in dicot and monocot plant species, since they are highly conserved across the plant kingdom [17,49,50]. However, the traditional overlapping PCR approach to construct amiRNA plasmid is tedious, time-consuming, and inefficient, especially for high-throughput application [17]. In this study, we provide a simplified method by mutating the sequence of pre-miR319a and pre-miR528 to create restriction sites for subsequent insertion of PCR products (Figure 1 and Figure 5). Therefore, the customized amiRNA/amiRNA* sequences can be easily inserted into the backbone of pre-miR319a and pre-miR528 by one single PCR, followed by restriction digestion and ligation. Our new strategy could dramatically improve the efficiency of amiRNA construction.

Many plant miRNAs such as Arabidopsis miR172a and miR169a are processed in a canonical “base to loop” manner [22,23,24]. For these miRNA precursors, the secondary structure of the lower stem is essential for miRNA processing. Disruption of the closing bulge structure in the lower stem by point mutation affects miRNA accumulation [23,24]. Meanwhile, other miRNAs such as Arabidopsis miR319a and miR159a have been reported to be processed in a “loop to base” direction [25]. Although complete removal of the lower stem sequences seemed to have little impact on the miR319 production, overexpressing the pre-miR319a lacking the lower stem caused a less severe leaf crinkled phenotype compared with overexpressing the full-length pre-miR319a [25]. We speculated that deletion of the lower stem bases may impair the accuracy of miR319a processing. Thus, full-length precursors are maintained as backbones for constructing engineered amiRNA vectors. In our case, the mutated sequences of the engineered pre-miR319a and pre-miR528 are located in the unpaired ssRNA region (Figure 1 and Figure 5), which may be less important for miRNA processing [25]. Indeed, the modifications on the pre-miR319a and pre-miR528 have little influence on their processing and functionality (Figure 2, Figure 3 and Figure 4 and Figure 6). It has been reported that miRNAs repress target gene expression through two modes of action, mRNA cleavage and translation inhibition [51]. However, the evaluation of efficacy of amiRNA in many studies is largely based on the measurement of target mRNA [45,46,47,48], without checking the abundance of target proteins. Using the ETPamir assay, we proved that amiRNAs produced by engineered pre-miR319a and pre-miR528 could effectively block the target protein accumulation (Figure 3 and Figure 6). The high efficacy of amiRNA expressed from the engineered pre-miR319a was further confirmed by the dwarf phenotype of amiR-*CNGC4*-1 overexpression lines (Figure 4). Taken together, we reason that the engineered amiRNA backbones should be fully functional as their original precursors.

In comparison to other amiRNA construction methods, our approach offers two advantages, as follows: First, full-length precursors are used as backbones and no changes are made in the lower stem, allowing amiRNAs to be processed accurately. Although some simplified amiRNA construction methods have been previously reported, those studies used the precursors either without the lower stem [47,52,53] or with a mutated lower stem [46,54,55] to express amiRNAs. Although the precursors in those methods could successfully produce amiRNAs and suppress target gene expression, at least in some cases there is no convincing evidence to prove that these truncated or mutated precursors are functioning equally like the full-length natural precursors. Second, we used the conventional restriction digestion-ligation strategy to construct amiRNA vectors, which balances the cloning efficiency and cost. Compared with the Gateway cloning system [56] and TA-based cloning system [52,54], whose cloning efficiency largely relies on commercial cloning kits or relatively expensive enzymes, our method is apparently more cost-saving.

In conclusion, we explored a simple and efficient method to construct amiRNA expression cassettes by creating restriction sites within the basal region of Arabidopsis and rice amiRNA precursors. We demonstrated that these modified amiRNA precursors are fully functional in plant protoplasts and transgenic plants. Hopefully, this new amiRNA cloning strategy will be useful for genome research in dicot and monocot plant species.

## 4. Materials and Methods

### 4.1. Plant Growth

Wild-type Col-0 or transgenic *Arabidopsis thaliana* plants were grown in a plant growth room on moistened Jiffy soil (Jiffy Substrates ^®^, Jiffy Group, Pärnumaa, Estonia), which are high-quality sphagnum peat-based growing substrates with a high organic content and water capacity to encourage rapid rooting and uniform growth. The Arabidopsis growth conditions are fixed at 65% humidity and 75 μmol·m^−2^·s^−1^ light intensity under photoperiods of 12 h light at 23 °C and 12 h dark at 20 °C. Zhonghua 11 rice (*Oryza sativa*) plants were grown on Jiffy soil in a plant growth chamber under photoperiods of 12 h light (200 μmol·m^−2^·s^−1^) at 30 °C and 12 h dark at 27 °C, with a constant humidity of 70%.

### 4.2. Plasmid Construction

Routine molecular cloning procedures were followed for plasmid construction. The original sequences of Arabidopsis miR319a or rice miR528 backbones were mutagenized by PCR-based mutagenesis to generate engineered miRNA precursors (pre-amiRNA). The amiRNA vectors *HBT-amiR-MEKK1*, *HBT-amiR-PDS3*, and *HBT-amiR-MAPKKK3* were constructed using pre-miR319a as the backbone, while *HBT-amiR-GFP* and *HBT-amiR-OsCEBiP* were constructed using pre-miR528 as the backbone. The original amiRNA vectors were cloned by traditional overlapping PCR, described by Schwab et al. [17]. The engineered amiRNA vectors were constructed as follows. Briefly, mega-primers containing customized amiRNA/amiRNA* sequences were used for PCR amplification of primary miRNA fragment containing a new amiRNA/amiRNA* duplex using the original pre-miR319a or pre-miR528 as PCR template. PCR amplicons were digested by *Eco*RI/*Xba*I or *StuI*/*Eco*RI and inserted into the same digested HBT vector harboring the engineered pre-miR319a or pre-miR528. For plant transformation, the pre-amiRNA was digested by *Bam*HI/*Pst*I and inserted into the same digested pCB302 binary vector.

To express a target gene encoding double HA- or FLAG-tagged target protein in protoplasts, the full-length coding sequences of target genes were amplified by RT-PCR, digested by *Bam*HI/*Stu*I and inserted into the same digested *HBT-2HA* or *HBT-2FLAG* vector, where the target gene expression is driven by the *35S* promoter.

### 4.3. Protoplast Isolation

Four-week-old Arabidopsis or 10-day-old *Oryza sativa* (Zhonghua 11 rice) seedlings were used for protoplast isolation according to the procedure described previously [32,57]. Briefly, leaves of Arabidopsis or sheaths of rice were cut into 0.5-mm strips with a sterile razor blade. The strips were digested in 10 mL enzyme solution (1.5% cellulase R10, 0.2% macerozyme R10, 0.4 M mannitol, 20 mM KCl, 20 mM MES, pH 5.7, 10 mM CaCl_2_, and 0.1% BSA) at room temperature for 3 h under a dark condition. After mixing with 10 mL W5 solution (154 mM NaCl, 125 mM CaCl_2_, 5 mM KCl, and 2 mM MES, pH 5.7), the digestion mixture was filtered through a 75 μm FALCON cell strainer. Protoplasts were collected by centrifugation in a CL2 clinical centrifuge (Thermo Scientific, Weaverville, North Carolina, USA) for 2 min at 100× *g* for Arabidopsis or 5 min at 200× *g* for rice. Cells were resuspended with 10 mL W5 solution and rested on ice for 30 min. Before transfection, protoplasts were pelleted by centrifugation for 1 min at 100× *g* for Arabidopsis or 3 min at 200× *g* for rice, and were then resuspended with MMg solution (0.4 M mannitol, 15 mM MgCl_2_, and 4 mM MES, pH 5.7) to a final concentration of 2 × 10^5^ cells per ml.

### 4.4. Protoplast Transfection and ETPamir Assay

DNA transfection was performed in a 2-mL round-bottom microcentrifuge tube, where 200 μL protoplasts were mixed with 21 μL (2 μg/μL) DNA cocktail and 220 μL PEG solution (40% PEG4000, *v*/*v*, 0.2 M mannitol and 0.1 M CaCl_2_), gently. After incubated at room temperature for 5 min (light) for Arabidopsis protoplasts or 15 min (dark) for rice protoplasts, transfection was quenched by adding 880 μL W5 solution. Transfected protoplasts were collected by centrifugation for 2 min at 100× *g* for Arabidopsis or 5 min at 200× *g* for rice, and were resuspended with 100 μL W5 solution. The cells were then transferred into 1 mL WI solution (0.5 M mannitol, 4 mM MES, pH 5.7, and 20 mM KCl) in a 6-well plate and were incubated in the dark.

The ETPamir assay was conducted according to the method described previously [31,32]. Briefly, 200 μL protoplasts were transfected with a DNA cocktail (2 μg/μL) containing 16 μL amiRNA expression construct, 4 μL target gene-HA/FLAG expression construct, and 1 μL transfection control plasmid expressing GFP-HA/FLAG. In parallel, a negative control was set up by replacing the amiRNA expression construct with an equal amount of empty vector. After co-transfection, protoplasts were incubated for 18–36 h in dark and then were collected for western blot analysis. The amiRNA performance was inversely correlated with the target protein accumulation.

### 4.5. Western Blot

After centrifugation, protoplasts were directly lysed with the lysis buffer (10 mM HEPES, pH 7.5, 100 mM NaCl, 1 mM EDTA, 10% Glycerol). The lysates were mixed with 6 × SDS-PAGE loading buffer and heated at 95 °C for 5 min or 55 °C for 10 min. Total proteins were subjected to SDS-PAGE (10%) and immunoblotting with anti-HA (Roche) or anti-FLAG (Sigma-Aldrich, Saint Louis, Missouri, USA) antibodies.

### 4.6. Generation and Screen of Transgenic Plants

The recombinant pCB302 binary plasmids were introduced into *Agrobacterium tumefaciens GV3101* cells by electroporation, which were in turn used for floral dip-mediated Arabidopsis transformation [58]. Transgenic Arabidopsis plants were selected on 1/2 MS medium containing 12.5 mg/L glufosinate ammonium.

### 4.7. RNA Extraction and Mature amiRNA Detection

For mature amiRNA detection in protoplasts, a total of 400 μL Arabidopsis protoplasts co-transfected with *HBT-pre-amiRNA* (original or engineered) plasmid and *pAN-mCherry-HA* plasmid were used for RNA extraction. For mature miR319a detection in transgenic plants, 30 mg rosette leaves of Col-0 and pre-miR319a overexpression lines were used for RNA extraction. Total RNA was extracted using the RNAiso Plus reagent (TaKaRa) according to the manufacturer’s instructions. The protocol described earlier [30] with minor modifications was used for mature amiRNA detection. Briefly, 1 μg total RNA was converted into the first-strand cDNA with stem-loop RT primers for amiRNA and gene specific primer of *mCherry* using a PrimeScript™ RT reagent Kit with genomic DNA Eraser (TaKaRa) according to the manufacturer’s instructions. RT-qPCR was performed in a LightCycler 96 Instrument (Roche) using the SYBR^®^ *Premix Ex Taq*^TM^ Kit (TaKaRa). Accumulation of mature amiRNAs produced from the original or engineered precursor in Arabidopsis protoplasts or pre-miR319a overexpression transgenic plants were normalized to the transcript levels of the transfection control *mCherry-HA* or *snoR101* (Small Nucleolar RNA 101), respectively.

## Supplementary Materials

Supplementary materials can be found at https://www.mdpi.com/1422-0067/20/22/5620/s1.

## Abbreviations

AS	Alternative splicing
amiRNA	Artificial microRNA
CRISPR	Clustered regularly interspaced short palindromic repeats
Cas9	CRISPR-associated protein 9
miRNA	MicroRNA
pre-miRNA	MiRNA precursor
pri-miRNA	Primary miRNA
T-DNA	Transfer DNA
PCR	Polymerase chain reaction
