# Supplementary material for: Engineered Artificial MicroRNA Precursors Facilitate Cloning and Gene Silencing in Arabidopsis and Rice
Source: Int J Mol Sci. 2019 Nov 10;20(22):5620. doi: 10.3390/ijms20225620 (PMC6888491; doi:10.3390/ijms20225620)
Supplement: Supplementary file 1 [file ijms-20-05620-s001.pdf]

Supplemental Table S1

Primers for amiRNA cloning with engineered pre-miR319a as backbone

Primer name	Primer sequences (5' to 3')
F-*EcoRI*	XXXGAATT**C**ATGTTTTAGGAATATATATGTAGA**NNNNNNNNNNNNNN** **NNNNNNN**TCACAGGTCGTGATATGATTC
R-*XbaI*	XXXTC**T**AGAAAATTGGAATACAAAAGAGAGA**NNNNNNNNNNNNNNNN** **NNNNN**TCAAAGAGAATCAATGATCCA

XXX: additional nucleotides to ensure efficient restriction digestion of PCR products.

N_{21}: capitalized nucleotides in Oligo III designed by WMD3 "Oligo" algorithm.

N_{21}: capitalized nucleotides in Oligo III designed by WMD3 "Oligo" algorithm.

> Arabidopsis pre-miR319a-based pre-amiRNA sequences

(The restriction sites of *Eco*RI and *Xba*I are unlined, and the modified sites are in bold. The amiRNA and amiRNA* highlighted in magenta and blue, respectively)

TCGAGCAAACACACGCTCGGACGCATATTACACATGTTCATACACTTAATACTCG
CTGTTTTGAATT**C**ATGTTTTAGGAATATATATGTAGAGGCATTTCCAACGTCCCTT
TTTCACAGGTCGTGATATGATTCAATTAGCTTCCGACTCATTCATCCAAATACCGA
GTCGCCAAAATTCAAACTAGACTCGTTAAATGAATGAATGATGCGGTAGACAAA
TTGGATCATTGATTCTCTTTGATAAAGGGTCGTTGGAAATACCTCTCTCTTTTGTA
TTCCAATTTTCT**A**GATTAATCTTTCCTGCACAAAAACATGCTTGATCCACTAAGTG
ACATATATGCTGCCTTCGTATATATAGTTCTGGTAAAATTAACATTTTGGGTTTAT
CTTTATTTAAGGCATCGCCATGACTAGT

Supplemental Table S2

Primers for amiRNA cloning with engineered pre-miR528 as backbone

Primer name	Primer sequences (5' to 3')
F-*StuI*	XXXAGG**C**CTGGTTTTTTGGCTGTAGCAGCAGCAG**NNNNNNNNNNNNN** **NNNNNNNN**CAGGAGATTCAGTTTGAAGCT
R-*EcoRI*	XXX**G**A**A**TTCCACAGAACAGCCTAGCAGCAGGAA**NNNNNNNNNNNNNN** **NNNNNNN**AGAGAGGCAAAAGTGAAGTCC

XXX: additional nucleotides to ensure efficient restriction digestion of PCR products.

N_{21}: capitalized nucleotides in Oligo I designed by WMD3 "Oligo" algorithm.

N_{21}: capitalized nucleotides in Oligo IV designed by WMD3 "Oligo" algorithm.

> Rice pre-miR528-based pre-amiRNA sequences

(The restriction sites of *Stu*I and *Eco*RI are unlined, and the modified sites are in bold. amiRNA and amiRNA* highlighted in magenta and blue, respectively)

CAGCAGCAGCCACAGCAAAATTTGGTTTGGGATAGGTAGGTGTTATGTTAGG**C**CT GGTTTTTTGGCTGTAGCAGCAGCAGTCCATAGGTGCCATCCGGGAGCAGGAGATT CAGTTTGAAGCTGGACTTCACTTTTGCCTCTCTCTCCCCGATCGCACCTATGGATT CCTGCTGCTAGGCTGTTCTGTGGAA**T**T**C**TGCAGAGTTTATATTATGGGTTTAATCG TCCATGGCATCAGCATCAGCAGCC
